# Dynamic changes in microbiota during fermentation of bamboo shoots under varying conditions in Guangxi, China

**DOI:** 10.3389/fmicb.2025.1529935

**Published:** 2025-04-16

**Authors:** JianWen Wu, Rong Qin, YanYan Lu, Gui Qing Li, Xing Fan

**Affiliations:** ^1^Guangxi Forestry Research Institute, Nanning, China; ^2^Guangxi Gaofeng State Owned Forest Farm, Nanning, China; ^3^Faculty of Agricultural Engineering, Guangxi Vocational and Technical College, Nanning, China

**Keywords:** *suansun*, traditional fermentation, modern fermentation, 16S rRNA, acid stress

## Abstract

**Introduction:**

The traditional fermentation process for bamboo shoots is long, complex, and yields a product with a non-uniform flavor. To address these issues, we determined the effects of using different parts of the bamboo shoot, different types of fermentation water, water-sealing or not, different types and initial concentrations of organic acids, and different lactobacillus inocula on microbial succession during the fermentation of bamboo shoots. 16S rRNA sequencing was performed using Illumina II high-throughput technology. Species abundance, *α*-, and *β*-diversity indices, and linear discriminant analysis effect size (LEfSe) analysis revealed that all detected microorganisms were members of the phyla Firmicutes, Cyanobacteria, and Proteobacteria, of which Firmicutes were dominant.

**Methods:**

The effects of these variables on microbial succession were evaluated through 16S rRNA sequencing using Illumina II high-throughput technology. Species abundance, *α*- and *β*-diversity indices, and linear discriminant analysis effect size (LEfSe) analysis were used to identify and analyze the microbial community.

**Results:**

The microbial community was dominated by the phyla Firmicutes, Cyanobacteria, and Proteobacteria, with Firmicutes being the most abundant. The relative abundance of Firmicutes was higher in water-sealed treatment groups than in non-sealed groups. Different organic acids selected specific microbial taxa, and the growth of acid-sensitive *Lactococcus* and *Weissella* was inhibited throughout fermentation. Different initial concentrations of organic acids selected biomarker taxa, such as *Sphingomonas*, which has the ability to degrade organic pollutants. When tap water was used as the fermentation broth, *Acinetobacter* became the dominant genus but inhibited the production of flavor compounds. *Streptococcus thermophilus* and *Lactobacillus bulgaricus*, derived from animal sources, had no significant effect on fermentation. Inoculation with a five-in-one lactic acid bacteria fermentation agent significantly increased the abundance of *Clostridium sensu stricto 1*, reaching a level 46.6 times that reported in the literature.

**Discussion:**

This study shows that various factors, including water-sealing, organic acids, and microbial inoculation, have significant effects on microbial succession and the flavor profile of fermented bamboo shoots. These findings suggest that optimizing these parameters can improve the consistency and flavor quality of the product.

## 1 Introduction

**Figure 1:**
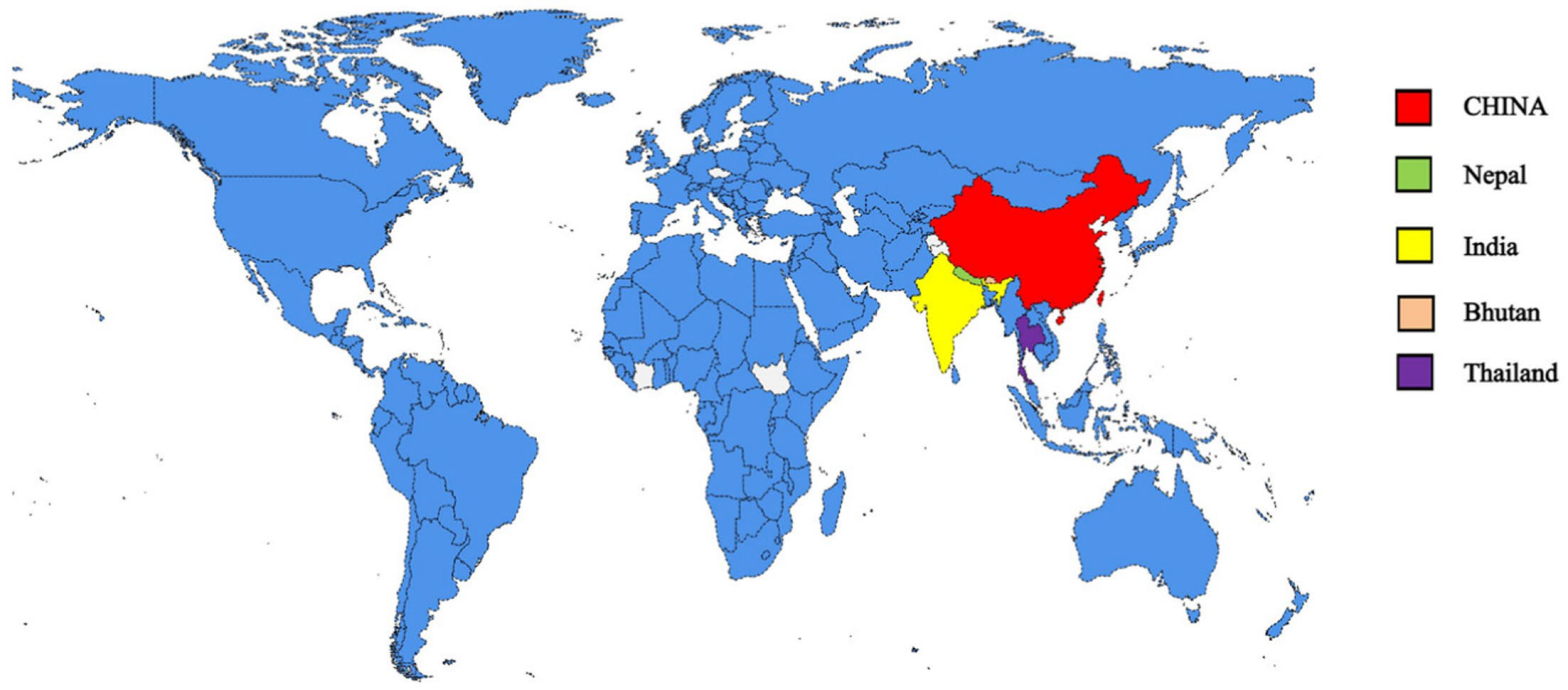
Global distribution of fermented bamboo-shoot production.

A total of 1,642 species of bamboo in 75 genera worldwide are recognized, of which more than 500 species in 39 genera are indigenous to China (Goyal et al., 2012; Goyal and Brahma, 2014; Choudhury et al., 2012; Wang et al., 2020). Raw, canned, boiled, marinated, fermented, frozen, liquid, and medicinal forms of bamboo shoots are all consumed. Around the world, a variety of fermented bamboo stalk products have been consumed. Ethnic populations in sub-Himalayan areas, comprising Nepal, Bhutan, Thailand, India, and China (Figure 1), produce fermented bamboo-shoot products known as *mesu*, *soibum*, *naw-mai-dong* or *nor-mai-dorng*, and *suansun*. *Mesu* is commonly used as a pickle and as a foundation for curries. A remarkable delicacy prepared by the Meitei people of Manipur, *soibum* is consumed as a pickle and in curries made with fermented fish (Choudhury et al., 2012). *Suansun* (sour bamboo shoots) is a primary ingredient of Liuzhou luosifen, and its unique “odor” and taste attracts consumers. In 2023, the sales revenue for the entire industry chain of Liuzhou luosifen in China amounted to 66.99 billion yuan, representing year-on-year growth of 11.5%. The “soul” of the Liuzhou luosifen flavor originates from *suansun* and the fermentation process is critical to the flavor. *Suansun* is prepared from fresh edible bamboo shoots, such as Ma bamboo (*Dendrocalamus latiflorus* Munro). The fresh bamboo shoots are peeled, cleaned, and chopped, then placed in pickling jars with either cold boiled water or mountain spring water to undergo spontaneous fermentation for 15–30 days at room temperature (Guan et al., 2020). *Lactobacillus* activity causes the bamboo shoots to gradually become sour during the pickling process. *Suansun* has the desirable characteristics of a crisp taste and sour flavor. However, fresh bamboo shoots are quickly lignified after harvest, which drastically lowers their commercial worth and food quality (Li J. et al., 2022; Zhang et al., 2020). The traditional natural fermentation cycle is long, overly dependent on experience, non-standardized, and far from able to meet the surging commercial demand. Therefore, it is important to conduct a comprehensive assessment of the crucial processes of bamboo shoot fermentation.

Irregular microbial succession and a difficult-to-control fermentation environment might result in variable quality. Indeed, multiple studies have reported an intricate link between complex microbial populations and the development of different taste components in fermented bamboo shoots. Substances such as *p*-cresol, lactic acid, acetic acid, and 1-octen-3-one contribute to the distinctive flavors created throughout the fermentation process (Hui et al., 2024). The critical stage in processing sour bamboo shoots is fermentation by soaking them in cold boiled water or mountain spring water for a specific period (Wang et al., 2020). To address the foregoing issues, researchers introduced inoculation fermentation. *Lactiplantibacillus plantarum* is among the most widely used strains in inoculation fermentation (Chen et al., 2024). Little is known about the succession and roles of these bacteria throughout the fermentation process, particularly the functional microorganisms responsible for sour flavor development. A comprehensive examination of the effects of the *suansun* fermentation process on the bacterial succession is important for the healthy development of the sour bamboo shoot industry. Famous Guangxi delicacies, such as Liuzhou luosifen, old friend noodles, Guilin rice noodles, and other traditional dishes, include pickled *suansun* as a supplementary ingredient. Especially during the COVID-19 pandemic, the popularity of Liuzhou luosifen as a domestic food dish surged. The daily average production of Liuzhou luosifen has reached 5.06 million packets. The pre-packaged products produced by industry are sold worldwide, which has led to a huge shortage in the supply of *suansun*. In this study, we examined the effects of different methods on the bacterial succession during sour bamboo shoots fermentation by focusing on the crucial factors of traditional and modern processes, comprising different parts of bamboo shoots, different types of water used for fermentation and processing, different sealing conditions, different organic acids and acid stress, as well as inoculation of different strains of bamboo shoots.

## 2 Materials and methods

### 2.1 Preparation and sampling of suansun

**Figure 2:**
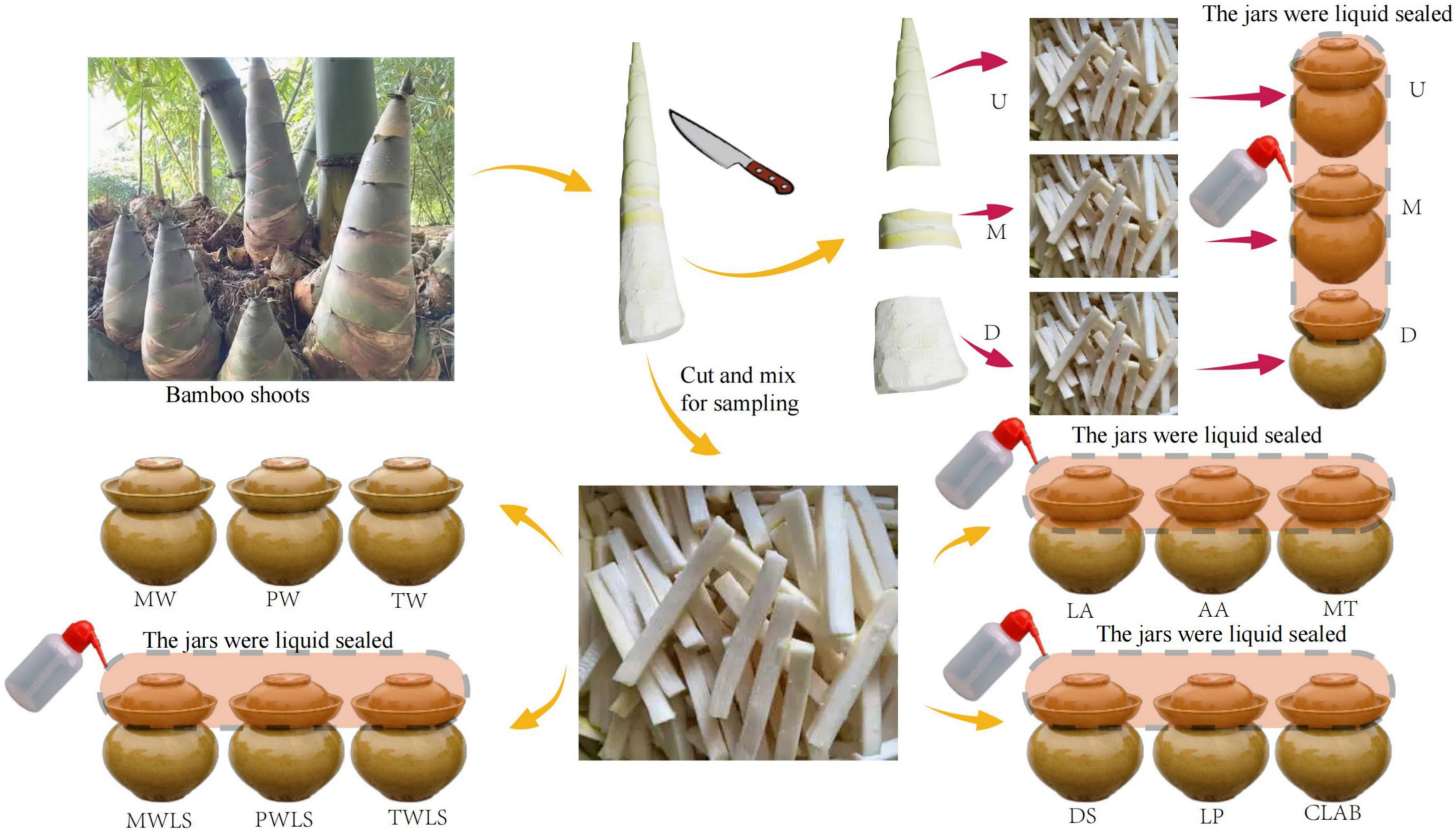
Schematic illustration of experimental design. Ceramic jars used for fermentation had a groove around the top, so the jars could be sealed by adding water. Three traditional fermentation methods were compared: liquid-sealed fermentation of the upper (U), middle (M), and lower (D) portions of bamboo shoots, and liquid-sealed and non-liquid-sealed fermentation with mountain spring water (MWLS, MW), pure water (PWLS, PW), or tap water (TWLS, TW). Two modern fermentation methods comprised different acid stress treatments [lactic acid (LA), acetic acid (AA), and mixed lactic acid–acetic acid (MT)] and inoculation with different bacterial fermentation inocula, namely, Danisco YO-MIX 883 (DS), *Lactobacillus plantarum* (LP), or five bacterial species (CLAB).

Fresh shoots of Ma bamboo (*Dendrocalamus latiflorus*) were harvested from Liucheng County, Liuzhou City, Guangxi Province, China, stored at 4°C, and transported to the laboratory within 24 h. The bamboo shoot shells were peeled, rinsed with sterile water, sliced into julienne strips 10 cm long, 0.5 cm wide, and 0.5 cm thick, and placed in 1 L ceramic jars for soaking in various water types. Each ceramic jar contained 800 g bamboo shoots, and each treatment had three replicates. The jars were divided into five groups with three treatments per group based on different bamboo shoot portions and water types used for processing. Each treatment was sampled on days 2, 8, 14, 20, 32, and 38 of fermentation for a total of 90 samples. At each time point, 15 unopened samples (one per treatment) were randomly selected for aseptic sampling and stored at −80°C for further analysis. The first group consisted of different portions of bamboo shoots, namely the upper (U), middle (M), and lower (D) portions, which were clearly demarcated from each other; U has bamboo shoot sheaths, with clear boundaries. And the layered bamboo shoot sheaths give the top of the bamboo shoots a unique appearance and taste. D is the hardest and most lignified portion, and M is intermediate in tenderness between U and D. The first group only uses purified water and undergoes liquid sealing treatment. The second group comprised three fermentation liquids, namely, mountain spring water (MW), purified water (PW), and tap water (TW), and the jars were not liquid sealed. The spring water was collected from Liucheng County, Liuzhou City, the main production area of *suansun*; the pure water was provided by Wahaha Group Co., Ltd. (Nanning, Guangxi Province, China); and the tap water was provided by Guangxi Nanning Water Co., Ltd. (Nanning, Guangxi Province, China). The third group was treated with liquid-sealed fermentation with each fermentation liquid, namely, mountain spring water (MWLS), purified water (PWLS), and tap water (TWLS). The second and third groups did not add organic acids or inoculants. The fourth group was only subjected to three organic acid stress treatments: 0.1 mol/L lactic acid (LA), 0.1 mol/L acetic acid (AA), and lactic acid–acetic acid mixture (MT). The concentrations of lactic acid and acetic acid in the mixed fermentation liquid were 11.59 and 3.57 g/L, respectively. The fifth group was inoculated with different bacterial fermenters, namely, Danisco YO-MIX™ 883 containing *Streptococcus thermophilus* and *Lactobacillus bulgaricus* (DS), a single *Lactobacillus plantarum* strain (LP), and a complex of lactobacilli comprising *Bifidobacterium lactis*, *Lactobacillus acidophilus*, *Lactobacillus casei*, *Lactobacillus rhamnosus*, and *Lactobacillus plantarum* (CLAB). The LP and CLAB strains were purchased from Weikai Haisi (Shandong) Bioengineering Co., Ltd. (Shandong, China). The inoculation amount was 2% of the mass of bamboo shoots. The fourth and fifth groups were all prepared with sterile water and sealed with liquid. The experimental design is illustrated in Figure 2. These processing methods mainly come from the folk fermentation methods in Liuzhou City, Guangxi Province.

### 2.2 DNA extraction

Total community genomic DNA was extracted using the E.Z.N.A™ MagBind Soil DNA Kit (M5635-02, Omega Bio-Tek, Norcross, GA, USA) following the manufacturer’s instructions. The DNA concentration was quantified using a Qubit 4.0 fluorometer (Thermo Fisher Scientific, Waltham, MA, USA) to ensure that adequate amounts of high-quality genomic DNA had been extracted.

### 2.3 16S rRNA gene amplification

The target was the V3–V4 hypervariable region of the bacterial 16S rRNA gene. The region was amplified by PCR immediately upon DNA extraction. The 16S rRNA V3–V4 fragment was amplified using two universal bacterial 16S rRNA gene amplicon PCR primers (PAGE purified): the amplicon PCR forward primer (CCTACGGGNGGCWGCAG) and amplicon PCR reverse primer (GACTACHVGGGTATCTAATCC). The reaction mixture composition was as follows: 2 μL microbial DNA (10 ng/μl), 1 μL amplicon PCR forward primer (10 μM), 1 μL amplicon PCR reverse primer (10 μM), and 30 μL 2× Hieff®Robust PCR Master Mix (Yeasen, 10105ES03, Shanghai, China). The plate was sealed and the PCR performed in a thermal-cycling instrument (Applied Biosystems 9,700, Foster City, CA, USA) using the following program: 1 cycle of denaturation at 95°C for 3 min; 5 cycles of denaturation at 95°C for 30 s, annealing at 45°C for 30 s, and elongation at 72°C for 30 s; 20 cycles of denaturation at 95°C for 30 s, annealing at 55°C for 30 s, and elongation at 72°C for 30 s; and a final extension at 72°C for 5 min. The PCR products were checked by electrophoresis in 2% (w/v) agarose gels in TBE buffer stained with ethidium bromide and visualized under ultraviolet light.

### 2.4 16S gene library construction, quantification, and sequencing

We used Hieff NGS™ DNA Selection Beads (Yeasen, 10105ES03) to purify the free primers and primer dimer species in the amplicon product. Samples were delivered to Sangon BioTech (Shanghai, China) for cDNA library construction using universal Illumina adaptors and indices. Before sequencing, the DNA concentration of each PCR product was determined using a Qubit® 4.0 Green double-stranded DNA assay and quality control was performed with a bioanalyzer (Agilent 2,100, Santa Clara, CA, USA). Depending on the coverage requirement, all libraries could be pooled for one run. The amplicons from each reaction mixture were pooled in equimolar ratios based on their concentration. Sequencing was performed using the Illumina MiSeq system (Illumina, San Diego, CA, USA) in accordance with the manufacturer’s instructions.

### 2.5 Sequence processing, OTU clustering, representative tags alignment, and taxonomic classification

After sequencing, the two short-read Illumina datasets were assembled with PEAR software (version 0.9.8) based on overlap in the short reads. The fastq files were processed to generate individual fasta and qual files, which could then be analyzed by standard methods. The effective tags were clustered into operational taxonomic units (OTUs) of ≥97% similarity using Usearch software (version 11.0.667). Chimeric sequences and singleton OTUs (with only one read) were removed, after which the remaining sequences were sorted into each sample based on the OTUs. The tag sequence with the highest abundance was selected as a representative sequence within each cluster. Bacterial and fungal OTU representative sequences were classified taxonomically by conducting a BLAST search against the RDP database and the UNITE database, respectively.

### 2.6 Statistical analysis

Alpha-diversity indices [comprising the Chao1, abundance-based coverage estimator (ACE), Simpson, and Shannon indices] were quantified as measures of OTU richness. To assess sample adequacy, rarefaction curves of the observed numbers of OTUs were constructed and all *α*-diversity indices were calculated with *Mothur* software (version 3.8.31). The OTU rarefaction curve and rank abundance curves were plotted in R (version 3.6.0). To estimate the diversity of the microbial community of the sample, we calculated the within-sample (α) diversity with a *t*-test for two groups and multiple group comparisons were performed using analysis of variance. Beta-diversity evaluates differences in the microbiome among samples and is normally combined with dimensional reduction methods, such as principal coordinate analysis (PCoA), non-metric multidimensional scaling, or constrained principal component analysis, to generate visual representations. The PCoA analysis results were visualized using the *vegan R package* (version 2.5–6) and the inter-sample distances were presented as scatterplots. Difference comparison was used to identify features with significantly different abundances between groups using *STAMP* (version 2.1.3) and *LEfSe* (version 1.1.0). Correlation coefficients and *p*-values between communities/OTUs were calculated using *SparCC* (version 1.1.0). Correlation matrix heatmaps were drawn using the *corrplot R package* (version 0.84). The *ggraph R package* (version 2.0.0) was used to construct network graphs.

## 3 Results

### 3.1 Microbial succession during suansun fermentation

**Figure 3:**
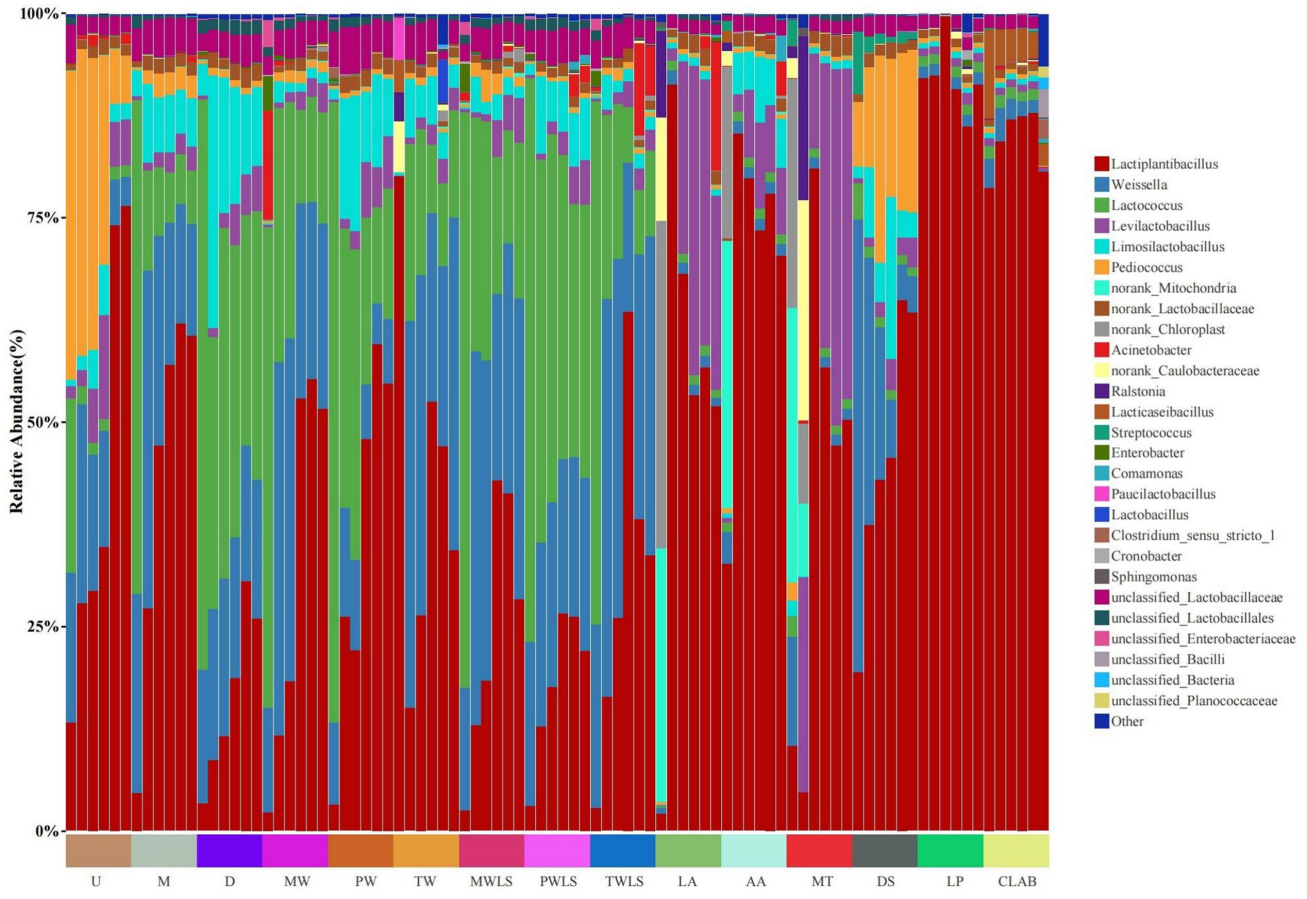
Changes in microbial community composition at the genus level during *suansun* fermentation. Three traditional fermentation methods, comprising liquid-sealed fermentation of the upper (U), middle (M), and lower (D) portions of bamboo shoots, and liquid-sealed and non-liquid-sealed fermentation with mountain spring water (MW), pure water (PW), or tap water (TW), were compared. Two modern fermentation methods comprised different acid stress treatments [lactic acid (LA), acetic acid (AA), and mixed lactic acid–acetic acid (MT)] and different bacterial inocula, namely, Danisco YO-MIX 883 (DS), *Lactobacillus plantarum* (LP), or five bacterial species (CLAB).

At the phylum level, Firmicutes abundance increased considerably following the onset of fermentation and was the dominant phylum at subsequent fermentation stages, with relative abundance exceeding 98.49% from day 2 of fermentation. At the genus level (Figure 3), *Pediococcus* (37.83%), *Lactococcus* (21.41%), *Weissella* (18.31%), and *Lactiplantibacillus* (13.24%) predominated in the U shoot portion on day 2 and decreased thereafter (except for *Lactiplantibacillus*, which showed 76.47% abundance at the conclusion of fermentation). The M and D shoot portions showed similar microbial successions, in that *Lactococcus* (60.49 and 69.76%, respectively) and *Weissella* (24.38 and 16.39%, respectively) predominated on day 2 and decreased in abundance thereafter, whereas *Lactiplantibacillus* abundance increased during fermentation to 60.64 and 25.94%, respectively.

Similar to MW, *Lactococcus* and *Weissella* predominated in MWLS on day 2 and subsequently decreased in abundance, and their relative abundances remained similar throughout fermentation, whereas *Lactiplantibacillus* became the predominant genus as fermentation progressed. The initial microbial flora compositions of PW and PWLS were similar, and the relative abundances of *Lactiplantibacillus*, *Weissella*, and *Lactococcus* were significantly more uniformly dispersed in PWLS than in PW when fermentation began on day 20 and continued through to day 38. Notably, *Lactiplantibacillus* in the TW treatment group increased considerably in abundance and became predominant on day 2; the relative abundance peaked at 80.13%, and decreased markedly to 34.33% by the end of the fermentation period. *Weissella* and *Lactococcus* increased from respective initial relative abundances of 0.019 and 0.029% to peak abundances of 47.34 and 21.65% on day 8, and decreased thereafter to 22.03 and 10.17% on day 32. Interestingly, the relative abundance composition of the main dominant flora in TW and TWLS was highly similar from the 8th day until the end of fermentation.

In the LA treatment, norank_Chloroplast (40.07%), norank_Mitochondria (31.09%), and norank_Caulobacteraceae (12.74%) predominated on day 2 and decreased remarkably to 0.085, 0.101, and 0.0058%, respectively, on day 8. From day 8, *Lactiplantibacillus* was predominant at the subsequent fermentation stages. Similar to LA, in the AA treatment from day 8, *Lactiplantibacillus* (85.38%) became predominant at the subsequent fermentation stages. *Lactiplantibacillus* (32.72%), norank_Mitochondria (32.70%), and norank_Chloroplast (20.97%) predominated on day 2, but norank_Mitochondria and norank_Chloroplast decreased in relative abundance to 0.20 and 0.15%, respectively, on day 8. The initial dominant genera in the MT treatment were *Lactiplantibacillus* (10.42%), *Weissella* (13.38%), norank_Mitochondria (33.67%), and norank_Chloroplast (28.10%). Ultimately, *Lactiplantibacillus* and *Levilactobacillus* became the dominant genera with relative abundances of 50.38 and 40.57%, respectively.

In both LP and CLAB, the dominant genus from the start of fermentation was *Lactiplantibacillus*. The DS treatment differed markedly in that *Lactiplantibacillus* and *Weissella* were the predominant strains on the second day of fermentation, and *Weissella* declined significantly in relative abundance as fermentation progressed to 7.16% on day 20. Notably, the relative abundance of *Pediococcus* increased gradually to a peak of 25.32% on day 14 of fermentation and subsequently decreased to 19.91% on day 20.

### 3.2 Alpha-diversity

**Figure 4:**
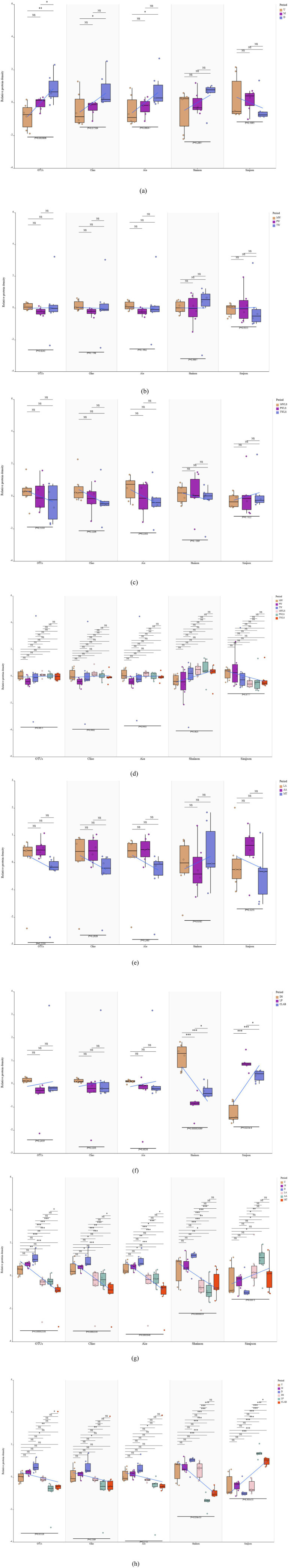
Chao1, abundance-based coverage estimator (ACE), Shannon, and Simpson indices of microbiota diversity in the different fermentation treatments. The horizontal bar within boxes represents the median. The upper and lower limits of the boxes represent the 75th and 25th percentiles, respectively. The upper and lower whiskers extend to data no more than 1.5× the interquartile range from the upper edge and lower edge of the box, respectively. The following comparisons of *α*-diversity among treatments are presented: **(a)** U, M, and D; **(b)** MW, PW, and TW; **(c)** MWLS, PWLS, and TWLS; **(d)** MW, PW, TW, MWLS, PWLS, and TWLS; **(e)** LA, AA, and MT; **(f)** DS, LP, and CLAB; **(g)** U, M, D, LA, AA, and MT; and **(h)** U, M, D, DS, LP, and CLAB. Three traditional fermentation methods, comprising liquid-sealed fermentation of the upper (U), middle (M), and lower (D) portions of bamboo shoots, and liquid-sealed and non-liquid-sealed fermentation with mountain spring water (MW), pure water (PW), or tap water (TW), were compared. Two modern fermentation methods comprised different acid stress treatments [lactic acid (LA), acetic acid (AA), and mixed lactic acid–acetic acid (MT)] and different bacterial inocula, namely Danisco YO-MIX 883 (DS), *Lactobacillus plantarum* (LP), or five bacterial species (CLAB).

The richness and diversity of the microbial communities were examined by analyzing the *α*-diversity (coverage rate > 99%) and calculating the Chao1, ACE, Shannon, and Simpson indices (Figure 4). The relative species abundance and bacterial diversity in the bamboo shoot portions both showed an increasing trend in the order U > M > D. The only significant difference in relative species abundance and bacterial diversity observed among the different bamboo shoot portions was of relative species abundance between U and D (Figure 4a). Regarding the different fermentation broths, the differences in *α*-diversity were not significant, regardless of the presence or absence of liquid sealing (Figures 4b–d). No differences in the α-diversity of samples among the organic acid stress treatments were significant (Figure 4e). Contrary to the trend observed for different bamboo shoot portions, the relative abundance of species indicated by the number of OTUs, Chao1, and ACE indices were not significantly affected by inoculation with different fermenters, whereas significant differences in the Shannon and Simpson indices were observed. Regarding the latter two indices, DS was highly significantly different from both LP and CLAB, and LP differed significantly from CLAB (Figure 4f). The bacterial diversity of CLAB was higher than that of LP, which reflected that LP was inoculated with a single *Lactobacillus plantarum* strain. Compared with U, M, and D under the traditional fermentation process, only the difference in species abundance between MT and U, M, and D among the organic acid stress treatments was highly significant; the difference in the Shannon index was significant, whereas the difference in the Simpson index was non-significant (Figure 4g). These results suggested that the source of variation in microbial diversity was concentrated among species of low relative abundance. The Shannon and Simpson indices of the LP and CLAB treatment groups were highly significantly different from those of the DS and the three bamboo shoot-portion treatment groups. In addition, for all of the *α*-diversity indices, DS did not differ significantly from the U, M, and D treatment groups (Figure 4h).

### 3.3 Beta-diversity

**Figure 5:**
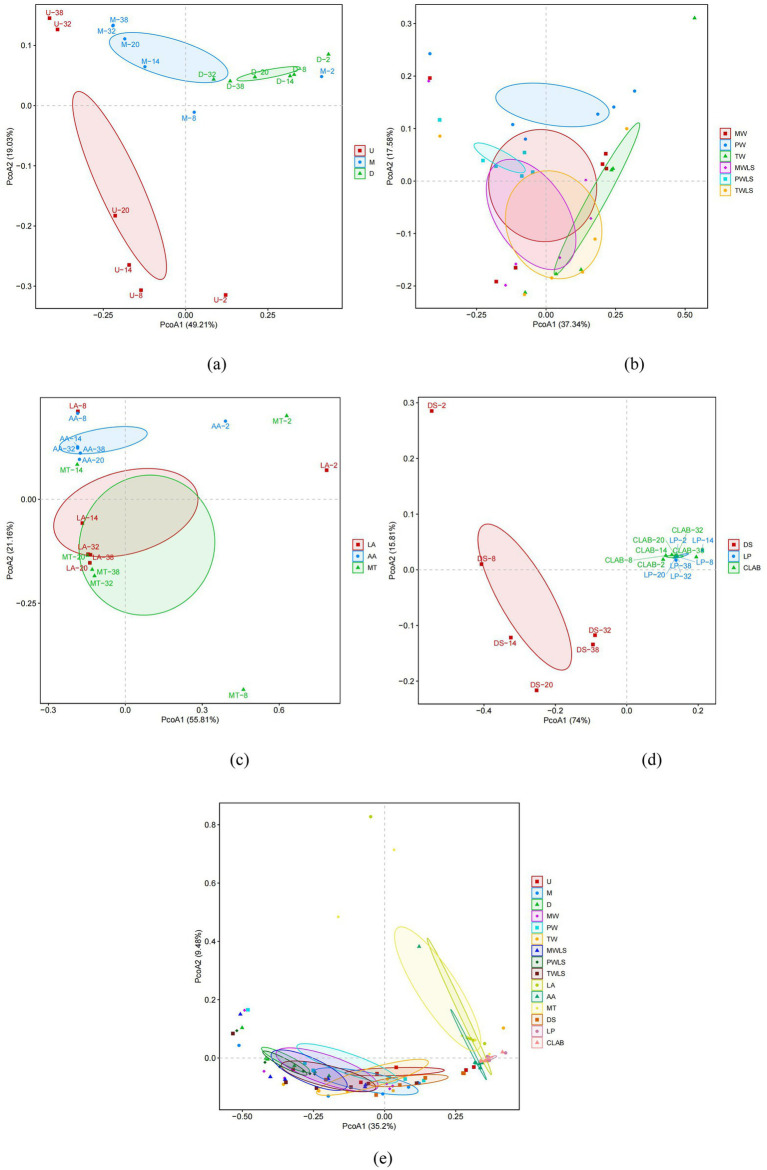
Unconstrained principal coordinate analysis (PCoA) of Bray–Curtis distances between the microbial communities in the different fermentation treatments. Results for the first and second principal coordinates (PCoA1 and PCoA2, respectively) are shown. The shaded elliptical regions represent 95% confidence intervals. The percentage of the total variation explained by each principal coordinate is shown in parentheses. Comparisons of treatment groups are presented as follows: **(a)** U, M, and D; **(b)** MW, PW, TW, MWLS, PWLS, and TWLS; **(c)** LA, AA, and MT; **(d)** DS, LP, and CLAB; and **(e)** the samples for all treatment groups. Three traditional fermentation methods, comprising liquid-sealed fermentation of the upper (U), middle (M), and lower (D) portions of bamboo shoots, and liquid-sealed and non-liquid-sealed fermentation with mountain spring water (MW), pure water (PW), or tap water (TW), were compared. Two modern fermentation methods comprised different acid stress treatments [lactic acid (LA), acetic acid (AA), and mixed lactic acid–acetic acid (MT)] and different bacterial inocula, namely, Danisco YO-MIX 883 (DS), *Lactobacillus plantarum* (LP), or five bacterial species (CLAB).

Unconstrained principal coordinate analysis (PCoA) of Bray–Curtis distances generally separated the modern and traditional fermentation techniques (except for the DS treatment group) into two separate clusters on the first principal coordinate, suggesting that the fermentation technique was the main source of variation in the microbiota of sour bamboo shoots (Figure 5e). The position of the DS samples was consistent with the aforementioned results of the α-diversity analysis. The microbiomes of the different bamboo shoot portions were separated into three distinct groups on both the first and second axes (Figure 5a). The different fermentation liquids and different organic acid stress treatments were all separated on the second principal coordinate, especially PW and AA (Figures 5b,c). The MW and MWLS treatment groups, and TW and TWLS groups, were not separated on either the first or second axes, consistent with the respective similarities in abundance and diversity of the microbial succession during fermentation (Figure 3). However, the PW and PWLS groups were separated on the second principal coordinate, which reflected the difference between liquid-sealed and non-liquid-sealed treatment. Among the MW, PW, TW, MWLS, PWLS, and TWLS treatment groups, only TW and PWLS were separated on the first axis. DS was clearly separated from LP and CLAB on the first axis, whereas LP and CLAB were not separated on the first and second axes (Figure 5d), which was consistent with the results of the *α*-diversity analysis (Figure 4f).

### 3.4 LEfSe diversity

**Figure 6:**
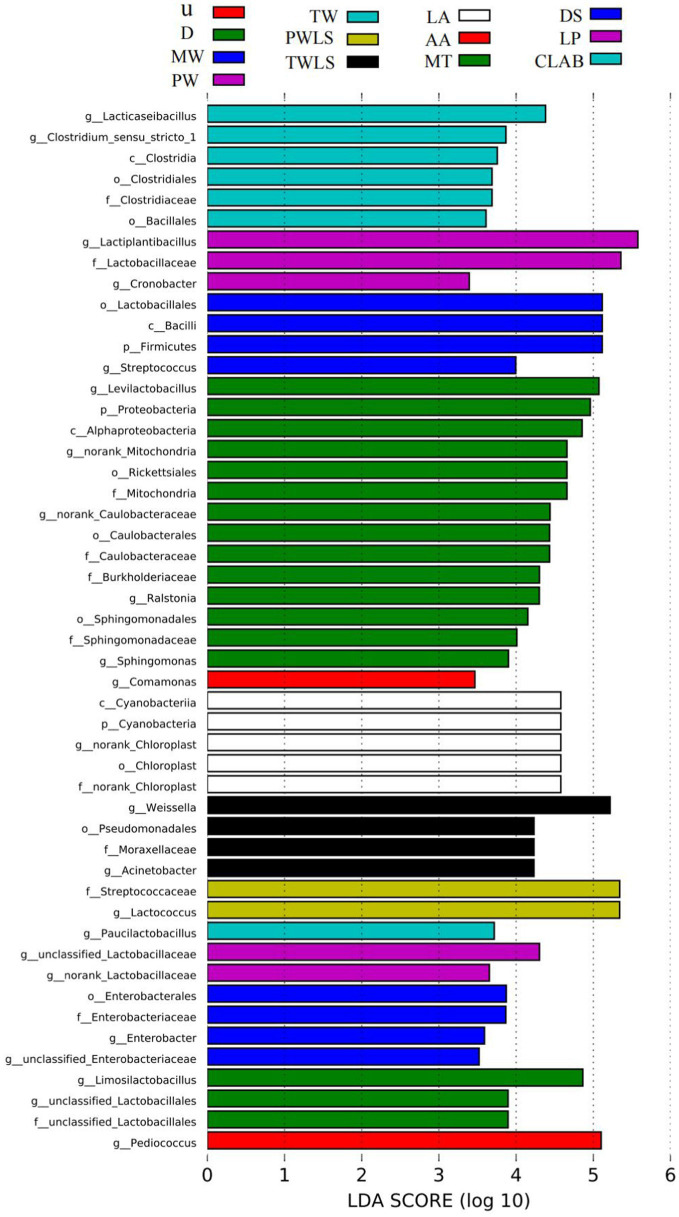
Indicator bacteria with linear discriminant analysis (LDA) scores ≥2 in bacterial communities associated with sour bamboo shoots under different fermentation treatments. Different colors represent different subgroups. The horizontal axis is the LDA score derived from the LDA analysis, and the vertical axis is the microbial taxa that are significantly enriched in the group represented by that color. Only results for LDA scores >2.0 are shown; the larger the value, the more strongly significant the difference. p, phylum; c, class; o, order; f, family; g, genus. Three traditional fermentation methods, comprising liquid-sealed fermentation of the upper (U), middle (M), and lower (D) portions of bamboo shoots, and liquid-sealed and non-liquid-sealed fermentation with mountain spring water (MW), pure water (PW), or tap water (TW), were compared. Two modern fermentation methods comprised different acid stress treatments [lactic acid (LA), acetic acid (AA), and mixed lactic acid–acetic acid (MT)] and different bacterial inocula, namely, Danisco YO-MIX 883 (DS), *Lactobacillus plantarum* (LP), or five bacterial species (CLAB).

**Figure 7:**
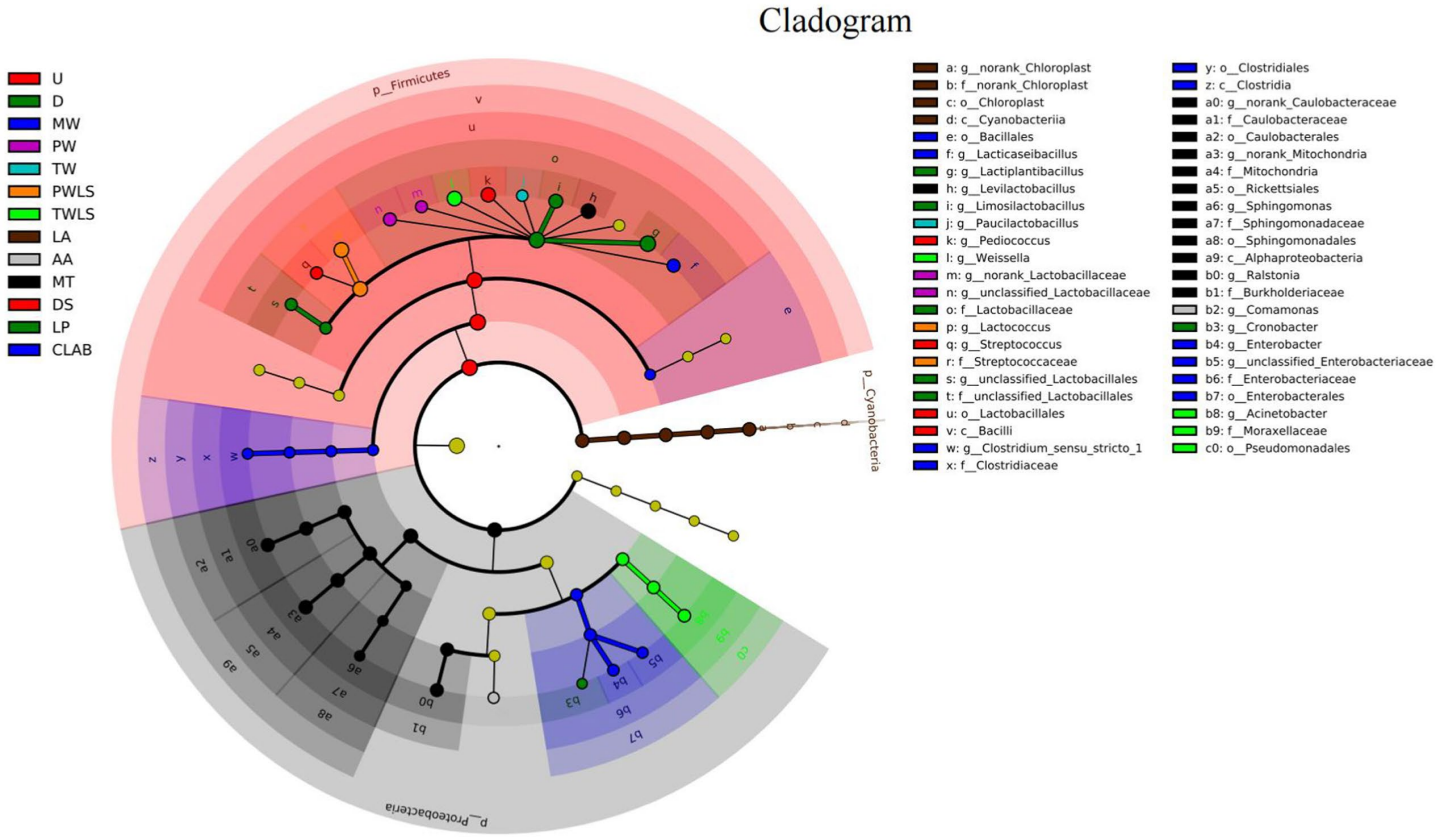
Cladogram showing the phylogenetic distribution of the bacterial lineages associated with sour bamboo shoots under different fermentation treatments. The cladogram was constructed from linear discriminant analysis (LDA) scores generated by a LDA effect size (LEfSe) analysis. From the innermost circle to the outermost circle, each node represents a phylum, class, order, family, and genus, respectively. Different colored circles at the branch nodes indicate microbial taxa that play an important role in the grouping corresponding to that color; yellow nodes indicate microorganisms that do not play an important role in any of the groups. The letters and numerals in the diagram represent different taxa; the specific names of the microorganisms are provided in the key on the right. p, phylum; c, class; o, order; f, family; g, genus. Three traditional fermentation methods, comprising liquid-sealed fermentation of the upper (U), middle (M), and lower (D) portions of bamboo shoots, and liquid-sealed and non-liquid-sealed fermentation with mountain spring water (MW), pure water (PW), or tap water (TW), were compared. Two modern fermentation methods comprised different acid stress treatments [lactic acid (LA), acetic acid (AA), and mixed lactic acid–acetic acid (MT)] and different bacterial inocula, namely, Danisco YO-MIX 883 (DS), *Lactobacillus plantarum* (LP), or five bacterial species (CLAB).

By identifying specialized bacterial communities of various groups, in addition to α- and *β*-diversity analysis, the community composition may be examined from a different perspective. We used the LEfSe software to search for biomarkers at the phylum to genus levels. Linear discriminant analysis (LDA) scores ≥2 were confirmed by LEfSe (Figure 6) and a cladogram derived from the LDA scores was constructed (Figure 7). The characteristic microorganisms for each fermentation mode were concentrated in three phyla: Firmicutes, Cyanobacteria, and Proteobacteria. The Proteobacteria and Cyanobacteria originate mainly from the surface or internal tissues of the raw materials (Guan et al., 2022). Firmicutes significantly increased in abundance and became predominant following the onset of fermentation. Significantly, Cyanobacteria comprised only norank_Chloroplast, o_Lactobacillales was the most highly enriched taxon in the Firmicutes (12 genera), and nine genera were enriched in the Proteobacteria. Specific details of the biomarkers for each treatment group are shown in Table 1. Modern fermentation techniques resulted in more species that were enriched at a substantial level (LDA >2) than under traditional fermentation techniques. Notably, MT had the most varied enriched biomarkers, comprising *Levilactobacillus*, norank_Caulobacteraceae, norank_Mitochondria, *Ralstonia*, and *Sphingomonas*, which are reported to be capable of degrading a wide range of organic pollutants, including biphenyls, naphthalenes, phenanthrene, dioxin-like compounds, carbazoles, chlorophenols, and a variety of herbicides and pesticides, and are able to withstand some of the most extreme environments (Maeda et al., 2003).

**Table 1:** Biomarkers and linear discriminant analysis (LDA) scores for each fermentation treatment group.

	Biomarker	LDA_score	Class
1	g.Pediococcus	5.10	U
2	g.unclassified.Lactobacillales	3.90	D
3	g.Limosilactobacillus	4.86	D
4	g.unclassified.Enterobacteriaceae	3.52	MW
5	g.Enterobacter	3.59	MW
6	g.unclassified.Lactobacillaceae	4.30	PW
7	g.norank.Lactobacillaceae	3.65	PW
8	g.Paucilactobacillus	3.72	TW
9	g.Lactococcus	5.34	PWLS
10	g.Acinetobacter	4.23	TWLS
11	g.Weissella	5.22	TWLS
12	g.norank.Chloroplast	4.58	LA
13	g.Comamonas	3.47	AA
14	g.Sphingomonas	3.90	MT
15	g.Levilactobacillus	5.07	MT
16	g.norank.Caulobacteraceae	4.44	MT
17	g.norank.Mitochondria	4.66	MT
18	g.Ralstonia	4.30	MT
19	g.Streptococcus	3.99	DS
20	g.Cronobacter	3.39	LP
21	g.Lactiplantibacillus	5.58	LP
22	g.Lacticaseibacillus	4.38	CLAB
23	g.Clostridium.sensu.stricto.1	3.87	CLAB

Three traditional fermentation methods, comprising liquid-sealed fermentation of the upper (U), middle (M), and lower (D) portions of bamboo shoots, and liquid-sealed and non-liquid-sealed fermentation with mountain spring water (MW), pure water (PW), or tap water (TW), were compared. Two modern fermentation methods comprised different acid stress treatments [lactic acid (LA), acetic acid (AA), and mixed lactic acid–acetic acid (MT)] and different bacterial inocula, namely, Danisco YO-MIX 883 (DS), *Lactobacillus plantarum* (LP), or five bacterial species (CLAB).

## 4 Discussion

Liuzhou luosifen, Guilin rice noodles, old friend noodles, snails and duck feet pot, and other famous Guangxi delicacies have been mass-produced through industrialization. Many are commonly available as pre-packaged foods, which have fueled consumer demand. *Suansun* is an important accompaniment to these dishes, but processes for its efficient industrialized production remain controversial. To address this controversy, we designed five groups of fermentation methods and monitored the successional changes in the bacterial population by 16S rRNA gene sequencing for a period of 38 days, with the aim to determine the production method and promote the development of the *suansun* industry. Detailed analysis of the changes in the microbial community of bamboo shoots under different fermentation conditions is crucial for industrialization of the method.

At the phylum level, from the second day of fermentation, the proportion of Firmicutes in the microbial community reached at least 99.12% (with relative abundance highest in U and lowest in D). These results are consistent with those of previous studies (Guan et al., 2022; Li Z. et al., 2022). The remainder of the microbial community comprised Proteobacteria and Cyanobacteria. Proteobacteria, Cyanobacteria, and Firmicutes account for approximately 82.85, 10.04, and 6.13%, respectively, of the bacterial community on fresh bamboo shoots (Li J. et al., 2022). Firmicutes and Proteobacteria were the dominant bacteria on fermented and fresh bamboo shoots, respectively. Proteobacteria abundance decreased rapidly after the start of fermentation. Alpha-diversity analysis showed that the diversity of communities in different portions of bamboo shoots did not differ significantly, and the relative abundance of species differed significantly only between U and D. The closer the shoot portion to the soil, the lower the relative abundance, but the higher the species uniformity. Given that U is the most tender portion of bamboo shoots, the difference in texture may be responsible for the highest relative abundance of Firmicutes. This finding parallels the research outcomes reported by Hu et al. (2023).

In the DS, LP, and CLAB treatments, also on the second day of fermentation, the percentage abundance of Firmicutes rose rapidly to more than 99.7%. Surprisingly, the percentage abundances of Proteobacteria, Cyanobacteria, and Firmicutes in LA on the second day of fermentation were 55.56, 40.08, and 3.98%, respectively, and it was not until the eighth day of fermentation that the percentage abundance of Firmicutes exceeded 99.7%. Similar to LA, in the AA treatment on the second day of fermentation, the percentage abundances of Proteobacteria, Cyanobacteria, and Firmicutes were 43.19, 35.80, and 20.97%, respectively, and only on the eighth day of fermentation did the percentage of Firmicutes reach more than 99.6%. Unusually, in MT the percentage of Firmicutes reached 99.8% on the 14th day of fermentation. Possibly because the different initial organic acids inhibited the growth of the acid-sensitive genera classified in the Firmicutes, a longer successional period was required for the abundance of Firmicutes to exceed 99%. The different organic acid stresses exerted a strong influence on the fermentation of bamboo shoots, laying a sound foundation for future in-depth studies.

Regarding the different fermentation liquids, the abundance of Firmicutes was higher in the liquid-sealed treatments than in the non-liquid-sealed treatments, and more especially in both PW treatment groups than in the MW and TW treatment groups. This result may be because mountain spring water and tap water introduce novel microbiota that compete with Firmicutes. However, the abundance of Firmicutes in all treatment groups in the present study never reached 99.99%, as reported previously by Guan et al. (2020). In summary, at the phylum level, fermentation after inoculation with different fermenters closely resembled the pattern of fermentation of different bamboo shoot portions without inoculation. Different initial organic acid treatments prolonged microbial succession, possibly lengthening the overall fermentation cycle, and provided a potential scenario for targeted stress screening of beneficial bacteria. The research results of Qin et al. (2024) indicate that these microorganisms have a major influence on the formation of organic acids in *suansun*. Pure water is the optimal choice for fermentation and liquid sealing improved the effectiveness of fermentation.

Succession of the major dominant microbial flora during *suansun* fermentation may be highly geographically dependent. According to Guan Q.’s analysis of all the flavorings in 47 *suansun* samples from 23 different regions, samples from the same region tended to cluster together using PCA analysis, while samples from other regions were well differentiated on the main coordinate axis. The *suansu*n samples from the closer-together regions of Guangdong, Guangxi, and Fujian had the most similar flavor compounds. Yunnan, which is farther away, had sour asparagus with more distinct flavor compounds than the other three places. *Lactococcus* and *Lactobacillus* are the two primary genera present during fermentation of sour bamboo shoots (*D. latiflorus*) in Yunnan (Caixia et al., 2022). *Weissella* initially predominates during the fermentation of sour bamboo shoots (*D. latiflorus*) in Guanxi, followed by *Lactobacillus*, which steadily increases to became the predominant bacterial genus as fermentation progresses (Li Z. et al., 2022).

Guan et al. reported that the accumulation of lactic and acetic acids causes the microbial flora to dynamically shift from the acid-sensitive species of *Lactococcus*, *Weissella*, and *Enterobacter* to the acid-resistant genus *Lactobacillus* (Guan et al., 2022). They concluded that the most important physicochemical factors affecting microbial succession are pH and titratable acidity. The accumulation of organic acids during fermentation of *suansun* leads to increasing acidity and decreasing pH. pH and titratable acidity gradients are the most important drivers of microbial succession, and the accumulation of lactic acid and acetic acid in the fermentation environment creates a specific acidic stress that inhibits the growth of microorganisms that are less acid-tolerant, resulting in the formation of a balanced microbial community dominated by acid-tolerant microorganisms of the genus *Lactobacillus*. These results are largely consistent with the findings of the present study. At the genus level, the dominant bacterial biota in fresh bamboo shoots were *Oxyphotobacteria* (unclassified) (10.04%) and *Lactobacillus* (5.29%). The proportion of *Lactococcus* in fresh bamboo shoots is 0.18% (Li J. et al., 2022).

In 2020, the International Commission on Bacteriological Nomenclature undertook a comprehensive review and update of the classification of wild forking bacteria. Among the most notable changes was the renaming of a portion of the genus *Lactobacillus* as *Lactiplantibacillus*. As the most common genus in the mid- to late-fermentation stages, *Lactobacillus* has a strong, positive association with the accumulation of the most distinctive flavor components, suggesting that it is primarily responsible for the flavor and taste development of *suansun* products. According to previous reports, *Lactobacillus* is responsible for production of volatile taste compounds and organic acids, particularly lactic acid, in a variety of fermented foods (Li Z. et al., 2022). These results are consistent with the present finding that *Lactiplantibacillus* eventually became the dominant genus in all treatment groups as fermentation progressed. A well-balanced microbial community that was dominated by the acid-resistant *Lactiplantibacillus* bacteria developed as a result of the buildup of lactic and acetic acids in the fermented environment, which produced a unique acidic stress that inhibited the growth of less acidic-tolerant microorganisms. This conclusion is verifiable from the results of the different initial organic acid treatment groups. The abundance of the acid-sensitive genera *Lactococcus* and *Weissella* was extremely low throughout fermentation, whereas their abundance in all other treatment groups decreased gradually, which reflected the impact of acidic stress.

Lactobacillus, Weissella, Lactococcus, Leuconostoc, Enterobacter, Raoultella, Kosakonia, Escherichia–Shigella, Acinetobacter, Clostridium sensu stricto 1, Peptoniphilus, and Acidocella were selected as core functional microorganisms (Guan et al., 2022). Enterobacter was a biomarker for MW (Table 1). Furthermore, the Enterobacter genus was previously thought to be a source of rot-causing bacteria in fermented vegetables (Franco and Pérez-Díaz, 2012). In the production area of Liuzhou luosifen, because of its flavor, the local residents prefer to use mountain spring water for pickling of bamboo shoots, but the need for liquid sealing has not been determined previously. To address this issue, we designed a two-group control experiment in which bamboo shoots were cured with mountain spring water, and the jars in one group were liquid-sealed with sterile water and those in the other group were not liquid-sealed. The microbial community succession and diversity analysis showed that the abundance and diversity of the predominant microbiota were extremely similar and did not differ significantly between the two treatment groups as fermentation progressed. Enterobacter reached an abundance of 4.19% on the second day of fermentation in MW, which was the highest abundance observed in all experimental groups. Therefore, liquid sealing had no significant impact on the fermentation of bamboo shoots in mountain spring water. However, from a biomarker perspective, the flavor of MW is expected to be superior, which will require verification in a subsequent flavor comparison. The observation that Lactiplantibacillus and Enterobacter are closely associated with the production of distinctive flavors provides a theoretical explanation for the selection of spring water for fermentation under the traditional process.

Acinetobacter, Enterobacter, Raoultella, Enterococcus, Klebsiella, Lactococcus, Leuconostoc, Weissella, Lactiplantibacillus, and Limosilactobacillus were considered to be the top 10 microbial genera associated with the unique flavor compounds of Guangxi bamboo shoots (Jian et al., 2024). These colonies are rich in enzymes that are crucial for flavor formation. Notably, p-cresol production in Guangxi bamboo shoots is attributed to Enterobacter, Raoultella, Klebsiella, Vibrio, and Acinetobacter. Enterobacter and Acinetobacter are the biomarkers listed in Table 1. Raoulella, Klebsiella, and Vibrio were also found, although their relative abundance was quite low. Therefore, we should additionally pay close attention to microbial colonies with low abundance response in the subsequent flavor analysis stage. Acinetobacter species, in contrast, were shown to have a strong but negative association with the majority of taste components, particularly volatile flavor compounds. This finding suggests that Acinetobacter may prevent the formation of flavor compounds during suansun fermentation. Acinetobacter is a type of spoilage bacteria that is frequently present in fish, shrimp, paocai, and soured milk (Li et al., 2018; Ribeiro Júnior et al., 2018; Wang et al., 2016; Zhu et al., 2018). Acinetobacter was a biomarker of the TWLS treatment group, which explains why we smelt an unpleasant pungent odor during the experiment. Thus, fermentation of suansun with tap water is not a recommended choice and presents a higher risk of failure.

The genus *Clostridium sensu stricto 1* exhibits a significant and positive correlation with *p*-cresol. Guan et al. (2022) suggested that this genus might be the most important contributor to the unique odor of *suansun*. Remarkably, however, *Clostridium sensu stricto 1* was not among the predominant genera associated with fermentation of *suansun*; instead, the relative abundance of this genus was extremely low (<0.05%) throughout the fermentation process (Guan et al., 2022). This finding was consistent, with the exception of two treatments, PW and CLAB. The relative abundance of *Clostridium sensu stricto 1* in PW was 1.24% on day 2 of fermentation, which was more than 24.8 times higher than that reported by previous studies, and decreased to <0.05% on day 20 of fermentation. LEfSe Diversity analysis showed that *Lactococcus* had the highest LDA score of 5.34 in PWLS samples. It is evident from Cladogram that only the biomarkers in the pure water treatment group are concentrated in Firmicutes. Thus, pure water is the most desirable of the three types of water used for processing. *Clostridium sensu stricto 1* was a biomarker for CLAB. It is notable that the abundance of *Clostridium sensu stricto 1* in CLAB at the end of fermentation was as high as 2.33%, and its relative abundance was more than 46.6 times higher than that reported in the literature. Therefore, to investigate the primary bacteria that contribute to the development of distinctive flavors, both dominance and functionality must be considered (Wang et al., 2016).

The biomarker for the DS treatment group was *Streptococcus*, which was strongly associated with the inoculation with *Streptococcus thermophilus* and *Lactobacillus bulgaricus*. It is very interesting to note that only *Weissella* abundance on the second day of DS fermentation was as high as 55.40%, whereas in the remainder of the treatment groups *Weissella* abundance was <24.38%. The predominant genera were *Lactiplantibacillus*, *Weissella*, *Limosilactobacillus*, and *Pediococcus*. *Limosilactobacillus* and *Pediococcus* were biomarkers for D and U, respectively. The *α*- and *β*-diversity analysis revealed that DS fermentation was not notably different from the traditional fermentation process. This indicates that inoculation of animal-derived lactic acid bacteria into plant materials for fermentation is not feasible, at least without clear evidence that it will improve the quality of the product. The LP treatment group was inoculated with a single *Lactobacillus plantarum* strain and *Lactiplantibacillus* was a biomarker for LP. From the second day of fermentation, the relative abundance of *Lactiplantibacillus* reached 92.13%, and on the 14th day of fermentation it was as high as 99.69%, with a more homogeneous diversity of genera. Therefore, the direct inoculation of favorable strains can stimulate the dominant bacterial colony to complete the succession in a short period, which is an effective means to achieve rapid fermentation of bamboo shoots for successful industrialization and standardization. More specifically, a number of *Lactobacillus* species, including *Lactobacillus plantarum*, *Lactobacillus fermentum*, and *Lactobacillus casei*, have been utilized as starter cultures and effectively employed to enhance the quality of the end products of numerous traditional fermented foods (Özer and Kılıç, 2020; Ruiz-Moyano et al., 2011).

Although statistically based indications of potential microbial biomarkers have provided many valuable insights into our research, it cannot be ignored that there are some drawbacks that deserve focused attention. On the one hand, the complexity and diversity of the fermentation process are revealed when faced with non-fermentor-driven fermentation scenarios. Due to the lack of uniform fermentation regulators, the characteristics of microbial communities are highly variable and may vary greatly depending on the specific fermentation process. Under such circumstances, it is not reasonable to judge or explore based on microbial biomarkers alone. This is because microbial biomarkers are often based on specific fermentation patterns or relatively stable microbial community structures, and in the non-fermentor-driven fermentation dynamic environment, it is difficult to accurately capture markers with real indicative significance, and the dynamic changes of microbial communities represented by these markers become elusive, which can easily lead to mislead the subsequent analysis and conclusion derivation. In summary, these drawbacks must be carefully considered when applying statistically based indicators of potential microbial biomarkers, especially when dealing with complex and variable research scenarios such as non-fermenter-driven fermentation, and incorporating more diversified research methodologies to ensure the accuracy and reliability of research results.

## 5 Conclusion

In the present study, *Dendrocalamus latiflorus* bamboo shoots from Liuzhou City, Guangxi, China, were used as the raw material for investigation of the microbial succession during five fermentation treatments for *suansun* production. The five fermentation processes were categorized into two types: the traditional fermentation process, which involved liquid-sealed fermentation of different portions of bamboo shoots, non-liquid-sealed fermentation with different processing water, and liquid-sealed fermentation with different processing water; and the modern fermentation process, which comprised liquid-sealed fermentation with stress from different organic acids and liquid-sealed fermentation with inoculation with different bacterial fermenters. All detected microorganisms were members of three phyla: Firmicutes, Cyanobacteria, and Proteobacteria. The predominant bacteria in fermented bamboo shoots were Firmicutes. Following fermentation, Proteobacteria dramatically decreased in abundance.

At the genus level, *Lactiplantibacillus* was ultimately the predominant genus in all treatment groups. The three portions of bamboo shoots showed significant differences in the relative abundance of bacterial taxa, but not in the diversity of the bacterial flora, during individual fermentation. *Pediococcus* was a biomarker for U. The succession patterns of the respective dominant flora in MW and MWLS, and in TW and TWLS, remained consistent, and neither the *α*- nor *β*-diversity differed significantly. Only the *β*-diversity of PW differed significantly from the other processed water groups, with a relative abundance of *Clostridium sensu stricto 1* of 1.24%, which was more than 24.8 times higher than that reported in the literature. *Acinetobacter* is a biomarker for TWLS that might inhibit the production of flavor compounds during *suansun* fermentation. Therefore, the use of tap water for fermentation of *suansun* should be avoided. The performance of purified water was more stable and liquid sealing effectively promoted fermentation. To obtain different flavors, spring water can be used for fermentation without liquid sealing. Incorporation of lactic acid, acetic acid, or a mixture of the two compounds in the water for fermentation and processing of bamboo shoots enables efficient screening of genera, and effectively inhibits acid-sensitive *Lactococcus* and *Weissella*. In addition, MT stress screened for *Sphingomonas*, which is able to tolerate various extreme environments and degrade a variety of organic pollutants, and only MT showed a highly significant difference in fermentation of U, M, and D under acid stress, with the source of the difference mainly attributable to bacterial species of low abundance. These results provide a sound foundation for future targeted screening of dominant bacterial genera.

With regard to introduction of bacterial strains, three schemes were designed in this study. Inoculation with *Streptococcus thermophilus* and *Lactobacillus bulgaricus* (DS) resulted in no differences from the traditional fermentation process. *Streptococcus thermophilus* and *Lactobacillus bulgaricus* from animal sources have no significant effect on the fermentation of bamboo shoots. Inoculation with a single *Lactobacillus plantarum* strain (LP) led to *Lactiplantibacillus* as the predominant genus throughout the fermentation process and the diversity of strains was undesirable, which was detrimental to the desirable sour asparagus flavor in the end product. Inoculation with a composite of five species, namely *Bifidobacterium lactis*, *Lactobacillus acidophilus*, *Lactobacillus casei*, *Lactobacillus rhamnosus*, and *Lactobacillus plantarum* resulted in *α*- and *β*-diversity significantly different from that of the conventional fermentation process. The abundance of *Clostridium sensu stricto 1*, a CLAB biomarker, was more than 46.6 times that reported in the literature. This genus is closely associated with the production of *p*-cresol, a flavor substance that is characteristic of sour asparagus.

In summary, varying the ratios of different portions of bamboo shoots can enrich the variety of the end products. It is easier to control the quality of the product by choosing aseptic purified water for fermentation. Adjustment of the type and concentration of organic acids in the fermentation broth enables targeted screening of the bacterial flora through acid stress. Inoculation with composite fermenter strains is an important means of achieving large-scale, industrialized production of *suansun*. Different acid stresses and their mechanisms are important topics for future research on *suansun* fermentation, and bacterial species of low abundance warrant greater attention. The introduction of complex lactic acid bacteria in different fermentation cycles could reduce the risk of fermentation failure and realize the targeted development of dominant bacterial succession. In addition, it may be possible to achieve self-clearing of harmful substances and enhance the flavor stability of the fermented products.

## Data Availability

The original contributions presented in the study are publicly available in the NCBI repository, accession numbers SRR32799332 - SRR32799346.
